# Association of obstructive sleep apnea with outcomes in hospitalized patients with pneumonia: a retrospective analysis of the nationwide inpatient sample

**DOI:** 10.1007/s11325-026-03855-0

**Published:** 2026-11-12

**Authors:** A B M Nasibul Alam, Blessing Chinyere Iheme, Oluwafunmbi Fatunmbi, Boluwaduro Adeyemi, Shaji Baig, Maryna Shayuk

**Affiliations:** 1 Internal Medicine Residency Program, Northwestern Medicine McHenry Hospital, Rosalind Franklin University of Medicine and Science, McHenry, IL 60050 USA; 2 Present Address: Pulmonary and Critical Care Medicine Fellowship Program, University of Tennessee Health Science Center, Knoxville, TN USA; 3 Department of Internal Medicine, American University of Barbados, Bridgetown, Barbados

**Keywords:** Obstructive sleep apnea, Pneumonia, Hospitalization, Acute respiratory failure, Nationwide Inpatient Sample, Mortality, Sepsis, Acute kidney injury

## Abstract

**Purpose:**

Obstructive sleep apnea (OSA) impairs mucociliary defense, reduces ventilatory reserve, promotes systemic inflammation, and predisposes patients to lower respiratory tract infections. Despite biological plausibility, the population-level association of comorbid OSA with pneumonia hospitalization outcomes remains poorly characterized. We aimed to evaluate associations between OSA and clinical outcomes in adults hospitalized with pneumonia using a nationally representative inpatient database.

**Methods:**

Using the 2018–2020 Nationwide Inpatient Sample (NIS), we conducted a retrospective cohort study of adults (≥ 18 years) hospitalized with a primary diagnosis of pneumonia, stratified by OSA status. Multivariable logistic and linear regression models adjusted for age, sex, race/ethnicity, household income quartile, primary payer, Charlson Comorbidity Index, obesity, hospital type, hospital bed size, and geographic region. P-values were corrected for multiple comparisons (Bonferroni), and E-values were calculated to assess sensitivity to unmeasured confounding.

**Results:**

Among 4,918,328 hospitalizations, 9.2% (*n* = 450,580) had coexisting OSA. Despite a greater comorbidity burden, OSA was associated with lower in-hospital mortality (3.3% vs. 4.8%; aOR 0.74; 95% CI 0.71–0.77; *p* < 0.0001), lower sepsis (aOR 0.89; 95% CI 0.87–0.90), and lower septic shock (2.2% vs. 2.7%; aOR 0.80; 95% CI 0.76–0.84). Conversely, OSA was associated with higher acute respiratory failure (48.1% vs. 35.8%; aOR 1.56; 95% CI 1.54–1.59), noninvasive ventilation (NIV) use (14.8% vs. 5.0%; aOR 3.26; 95% CI 3.17–3.34), and acute kidney injury (AKI; 24.1% vs. 22.1%; aOR 1.13; 95% CI 1.11–1.15). Adjusted length of stay was slightly longer (β + 0.11 days; 95% CI 0.07–0.16), while adjusted hospital charges did not differ significantly. All associations except hospital charges remained significant after correction for multiple comparisons; E-values suggested that the sepsis and AKI associations could be explained by relatively weak unmeasured confounding.

**Conclusions:**

Among adults hospitalized with pneumonia, comorbid OSA was associated with higher rates of acute respiratory failure, NIV use, and AKI, and with lower in-hospital mortality and sepsis. Because of the retrospective administrative design and the lack of data on pneumonia severity, OSA severity, and CPAP use, residual and unmeasured confounding cannot be excluded. These findings are hypothesis-generating, and prospective studies are needed.

**Supplementary Information:**

The online version contains supplementary material available at https://doi.org/10.1007/s11325-026-03855-0.

## Introduction

Obstructive sleep apnea (OSA) is among the most prevalent chronic respiratory disorders worldwide, estimated to affect approximately 936 million adults aged 30–69 years globally, of whom 425 million have moderate-to-severe disease requiring treatment [1, 2]. In the United States, an estimated 23.5 million individuals with OSA remain undiagnosed [3], creating a substantial reservoir of physiologically vulnerable patients who present to hospital without a known sleep-disordered breathing diagnosis. The disorder is characterized by recurrent episodes of partial or complete upper airway obstruction during sleep that generate cyclical intermittent hypoxia (IH), hypercapnia, autonomic dysregulation, and fragmentation of restorative sleep architecture. These nightly events activate NF-κB and HIF-1α signaling, generate reactive oxygen species, suppress type I interferon responses, and sustain a state of chronic low-grade systemic inflammation characterized by elevated circulating IL-6, TNF-α, and C-reactive protein [4]. The downstream cardiovascular, metabolic, and neurological consequences are well-established: OSA is associated with increased risk of hypertension, atrial fibrillation, heart failure, type 2 diabetes, stroke, and all-cause mortality, with severe OSA carrying an adjusted hazard ratio for all-cause mortality of 2.13 (95% CI 1.68–2.68) [4, 5].

Acute pneumonia represents a particularly hazardous clinical intersection with OSA. Multiple converging pathophysiological pathways may amplify both susceptibility and severity: (1) repeated upper airway collapse and coexisting gastroesophageal reflux facilitate microaspiration of oropharyngeal flora [6]; (2) OSA disrupts the upper airway microbiome and impairs mucociliary clearance, promoting pathogen colonization [7]; (3) chronic IH blunts hypercapnic and hypoxic ventilatory responses, reducing the reserve available to compensate for acute parenchymal infection [8]; and (4) dysregulation of innate immunity — including reduced neutrophil phagocytic capacity, altered macrophage polarization, and suppressed type I interferon signaling — may compromise pulmonary host defense [9, 10]. Chiner et al. reported that OSA was independently associated with a 2.86-fold increased risk of community-acquired pneumonia (OR 2.86; 95% CI 1.29–6.44) and that OSA severity independently predicted pneumonia severity [6]. A 2024 narrative review summarized reports of elevated rates of invasive pneumococcal disease and bacterial pneumonia in patients with OSA [11]. Despite this biological plausibility, population-level evidence on OSA–pneumonia inpatient outcomes remains limited, with prior studies restricted by small sample sizes or exclusive focus on COVID-19 pneumonia (meta-analysis OR for mortality 1.74; 95% CI 1.39–2.19) [12]. This study addresses that gap by leveraging the 2018–2020 Nationwide Inpatient Sample (NIS) to characterize associations between comorbid OSA and in-hospital mortality, length of stay, hospital charges, acute respiratory failure, ventilatory support, AKI, sepsis, and septic shock in adults hospitalized with pneumonia.

## Methods

### Data source and study population

This retrospective cohort study utilized data from the NIS for 2018–2020. The NIS is the largest publicly available all-payer inpatient database in the United States, capturing patient demographics, comorbidities, procedures, and outcomes from diverse hospitals nationwide. Adults (≥ 18 years) with a primary discharge diagnosis of pneumonia (ICD-10-CM J12–J18) were included. Patients receiving palliative or hospice care and those with missing demographic data were excluded. The study population was stratified by the presence of a secondary diagnosis of OSA (ICD-10-CM G47.33).

### Outcomes

The primary outcome was in-hospital mortality. Secondary outcomes included length of stay (LOS), total hospital charges, acute kidney injury (AKI), sepsis, septic shock, acute respiratory failure, noninvasive ventilation (NIV) use, and invasive mechanical ventilation (MV) use, all identified by ICD-10-CM secondary diagnosis or procedure codes.

### Statistical analysis

Categorical variables were reported as weighted frequencies and percentages and compared using chi-square tests. Continuous variables were reported as weighted means ± SE and compared using independent-samples t-tests. Length of stay and hospital charges are right-skewed; however, formal tests of normality are uninformative at this sample size, and comparisons of means in nearly five million discharges are valid because the sampling distribution of the mean is approximately normal by the central limit theorem. Adjusted differences in continuous outcomes were estimated by survey-weighted linear regression. Binary outcomes were analyzed using multivariable logistic regression reporting adjusted odds ratios (aOR) with 95% confidence intervals (CI). Continuous outcomes were analyzed using linear regression. All models adjusted for age, sex, race/ethnicity, household income quartile, primary payer, Charlson Comorbidity Index, obesity, hospital type, hospital bed size, and geographic region. Obesity was identified using ICD-10-CM E66 codes (Supplementary Table S1). Hospital bed size refers to hospital bed capacity, categorized as small, medium, or large according to HCUP definitions, which vary by hospital region, location (rural/urban), and teaching status. A two-tailed *p* < 0.05 was considered statistically significant. All analyses applied NIS discharge weights to produce nationally representative estimates.

Because nine outcomes were examined, we applied a Bonferroni correction (adjusted significance threshold 0.05/9 = 0.0056). To assess sensitivity to unmeasured confounding, we calculated E-values [13] for the adjusted odds ratios and the confidence limits closest to the null. Odds ratios were treated as approximate risk ratios for outcomes with prevalence below 10% in the non-OSA group (mortality, septic shock, NIV, MV) and converted using the square-root approximation for more common outcomes (sepsis, AKI, acute respiratory failure).

## Results

### Study population and temporal distribution

A total of 4,467,748 pneumonia patients without OSA and 450,580 with OSA were included. Hospitalizations increased across the study period, with the largest proportion in 2020 (37.0% non-OSA; 36.5% OSA; *p* < 0.0001) (Table 1).

**Table 1:** Distribution of pneumonia hospitalizations across 2018–2020 (NIS time trends)

Variable	Pneumonia without OSA (weighted *n* = 4,467,748)		Pneumonia with OSA (weighted *n* = 450,580)		*P* value
	Weighted *N*	Col %	Weighted *N*	Col %	
NIS 2018	1,420,414	31.8%	139,595	31.0%	< 0.0001
NIS 2019	1,392,039	31.2%	146,310	32.5%	< 0.0001
NIS 2020	1,655,295	37.0%	164,675	36.5%	< 0.0001

*OSA *Obstructive sleep apnea, *NIS *Nationwide Inpatient Sample

### Patient characteristics

OSA patients were slightly younger (mean age 66 vs. 68 years; *p* < 0.0001), more often male (56.7% vs. 48.7%), and more likely to be White (74.2% vs. 68.4%). OSA patients were somewhat more frequently treated in urban teaching hospitals (65.9% vs. 61.8%), and Medicare was the predominant payer in both groups (Table 2). OSA patients had a greater comorbidity burden: 98.5% had a Charlson Comorbidity Index ≥ 3 (vs. 94.9%); obesity was more prevalent (47.1% vs. 14.0%), as were type 2 diabetes mellitus (26.2% vs. 16.1%), chronic kidney disease (29.8% vs. 21.8%), and chronic heart failure (34.9% vs. 21.9%); all *p* < 0.0001 (Table 3). Given the sample size, several statistically significant baseline differences were small in absolute magnitude (e.g., age, hospital type, and Charlson Comorbidity Index category).

**Table 2:** Baseline patient characteristics stratified by OSA status

Variable	Pneumonia without OSA (*n* = 4,467,748)		Pneumonia with OSA (*n* = 450,580)		*P* value
	Mean (SE) / *N*	%	Mean (SE) / *N*	%	
Age, years (mean ± SE)	68 (0.0)	—	66 (0.1)	—	< 0.0001
Age group					< 0.0001
18–30	146,245	3.3	5,810	1.3	
31–40	203,065	4.5	14,145	3.1	
41–50	320,290	7.2	35,520	7.9	
51–60	644,630	14.4	80,960	18.0	
61–70	946,285	21.2	123,535	27.4	
71–80	1,056,845	23.7	124,300	27.6	
>80	1,150,389	25.7	66,310	14.7	
Race/Ethnicity					< 0.0001
White	3,053,754	68.4	334,480	74.2	
Black	588,525	13.2	58,920	13.1	
Hispanic	462,635	10.4	29,775	6.6	
Asian/Pacific Islander	112,945	2.5	6,210	1.4	
Native American	33,585	0.8	2,855	0.6	
Other	216,305	4.8	18,340	4.1	
Sex					< 0.0001
Male	2,175,689	48.7	255,420	56.7	
Female	2,291,879	51.3	195,140	43.3	
Hospital region					< 0.0001
Northeast	773,804	17.3	68,860	15.3	
Midwest	898,240	20.1	126,790	28.1	
South	1,977,781	44.3	179,485	39.8	
West	817,923	18.3	75,445	16.7	
Hospital type					< 0.0001
Rural	690,584	15.5	58,065	12.9	
Urban nonteaching	1,016,130	22.7	95,450	21.2	
Urban teaching	2,761,034	61.8	297,065	65.9	
Hospital bed size					< 0.0001
Small	1,193,752	26.7	110,510	24.5	
Medium	1,359,244	30.4	134,825	29.9	
Large	1,914,752	42.9	205,245	45.6	
Primary payer					< 0.0001
Medicare	2,887,264	64.6	302,230	67.1	
Medicaid	501,040	11.2	39,645	8.8	
Private insurance	789,630	17.7	88,320	19.6	
Self-pay	158,740	3.6	7,885	1.7	
No charge	125,695	2.8	12,040	2.7	
Other	5,380	0.1	460	0.1	
Household income					< 0.0001
Q1 (lowest)	1,558,364	34.9	139,845	31.0	
Q2	1,213,240	27.2	127,865	28.4	
Q3	957,390	21.4	106,585	23.7	
Q4 (highest)	738,755	16.5	76,285	16.9	

All p values derived from chi-square (categorical) or independent-samples t-test (continuous). Q1–Q4 = household income quartile (Q1 lowest, Q4 highest). Hospital bed size = hospital bed capacity category (small, medium, large) as defined by HCUP

**Table 3:** Comorbidity profile of pneumonia patients stratified by OSA status

Comorbidity	Pneumonia without OSA (*n* = 4,467,748)		Pneumonia with OSA (*n* = 450,580)		*P* value
	*N* (weighted)	%	*N* (weighted)	%	
CCI category					< 0.0001
CCI = 1	88,985	2.0	1,730	0.4	
CCI = 2	140,295	3.1	5,060	1.1	
CCI ≥ 3	4,238,468	94.9	443,790	98.5	
COPD	413,145	9.2	47,880	10.6	< 0.0001
Type 2 diabetes mellitus	717,670	16.1	118,165	26.2	< 0.0001
Chronic kidney disease	975,335	21.8	134,140	29.8	< 0.0001
Chronic heart failure	979,665	21.9	157,185	34.9	< 0.0001
Obesityᵃ	625,485	14.0	212,045	47.1	< 0.0001
Essential hypertension	1,541,679	34.5	146,945	32.6	< 0.0001

*CKD *Chronic kidney disease, *COPD *Chronic obstructive pulmonary disease, *CCI *Charlson Comorbidity Index

ᵃWeighted N for the non-OSA group was calculated from the reported prevalence (14.0%)

### Primary outcome: in-hospital mortality

In-hospital mortality was lower in OSA patients (3.3% vs. 4.8%; absolute difference 1.5% points; *p* < 0.0001). The unadjusted model showed 32% lower odds of death (OR 0.68; 95% CI 0.65–0.70). After multivariable adjustment, the association persisted, with 26% lower odds of in-hospital death (aOR 0.74; 95% CI 0.71–0.77; *p* < 0.0001) (Table 5).

### Length of stay and hospital charges

Mean LOS was slightly longer in OSA patients (6.00 vs. 5.83 days; *p* < 0.0001; adjusted β + 0.11 days; 95% CI 0.07–0.16). Mean hospital charges were slightly higher ($62,946 vs. $61,282); however, after adjustment this difference was not statistically significant (adjusted difference $598.83; 95% CI −$90.16 to $1,287.82; *p* = 0.088) (Table 4).

**Table 4:** Clinical outcomes: length of stay and hospital charges

Outcome	Pneumonia without OSA (*n* = 4,467,748)		Pneumonia with OSA (*n* = 450,580)		Crude coefficient (95% CI)	*p*	Adjusted coefficient* (95% CI)	*p*
	Mean	SE	Mean	SE				
Length of stay (days)	5.83	0.015	6.00	0.024	0.17 (0.13, 0.22)	< 0.0001	0.11 (0.07, 0.16)	< 0.0001
Total hospital charge (USD)	$61,282	$438	$62,946	$504	$1,665 ($972, $2,357)	< 0.0001	$599 (−$90, $1,288)	0.088

*CI *Confidence interval, *SE *Standard error, *USD *US dollars

*Adjusted for age, sex, race/ethnicity, Charlson Comorbidity Index, obesity, household income, primary payer, hospital type, bed size, and geographic region

### Respiratory complications and ventilatory support

Acute respiratory failure occurred in 48.1% of OSA patients versus 35.8% of non-OSA patients (aOR 1.56; 95% CI 1.54–1.59; *p* < 0.0001). NIV use was approximately threefold higher (14.8% vs. 5.0%; aOR 3.26; 95% CI 3.17–3.34) and MV use was slightly more frequent (6.3% vs. 5.4%; aOR 1.11; 95% CI 1.09–1.13).

### Systemic complications

AKI occurred more frequently in OSA patients (24.1% vs. 22.1%; aOR 1.13; 95% CI 1.11–1.15; *p* < 0.0001), a difference of 2.0% points. Sepsis (19.6% vs. 21.6%; aOR 0.89; 95% CI 0.87–0.90) and septic shock (2.2% vs. 2.7%; aOR 0.80; 95% CI 0.76–0.84) were both lower in OSA patients (all *p* < 0.0001), with absolute differences of 2.0 and 0.5% points, respectively (Table 5).

**Table 5:** Clinical outcomes: binary endpoints — unadjusted and adjusted odds ratios

Outcome	Pneumonia without OSA (*n* = 4,467,748)		Pneumonia with OSA (*n* = 450,580)		Unadjusted OR (95% CI)	*p*	aOR* (95% CI)	*p*
	*N* (weighted)	%	*N* (weighted)	%				
In-hospital mortality	213,305	4.8	14,805	3.3	0.68 (0.65, 0.70)	< 0.0001	0.74 (0.71, 0.77)	< 0.0001
AKI	987,690	22.1	108,815	24.1	1.12 (1.10, 1.14)	< 0.0001	1.13 (1.11, 1.15)	< 0.0001
Sepsis	966,845	21.6	88,385	19.6	0.88 (0.87, 0.90)	< 0.0001	0.89 (0.87, 0.90)	< 0.0001
Septic shock	118,510	2.7	9,885	2.2	0.82 (0.78, 0.86)	< 0.0001	0.80 (0.76, 0.84)	< 0.0001
Acute respiratory failure	1,597,515	35.8	216,715	48.1	1.66 (1.64, 1.69)	< 0.0001	1.56 (1.54, 1.59)	< 0.0001
NIV	223,750	5.0	66,510	14.8	3.28 (3.20, 3.37)	< 0.0001	3.26 (3.17, 3.34)	< 0.0001
MV	241,325	5.4	28,450	6.3	1.18 (1.14, 1.22)	< 0.0001	1.11 (1.09, 1.13)	< 0.0001

*aOR *Adjusted odds ratio, *CI *Confidence interval, *AKI *Acute kidney injury, *NIV *Noninvasive ventilation, *MV *Mechanical ventilation

*Adjusted for age, sex, race/ethnicity, Charlson Comorbidity Index, obesity, household income, primary payer, hospital type, bed size, and geographic region

### Multiple comparisons and sensitivity to unmeasured confounding

After Bonferroni correction (threshold *p* < 0.0056), all associations remained statistically significant except adjusted hospital charges (*p* = 0.088). E-values (point estimate; confidence limit closest to the null) were 2.04 (1.92) for mortality, 1.81 (1.67) for septic shock, 1.31 (1.29) for sepsis, 1.32 (1.29) for AKI, 1.81 (1.79) for acute respiratory failure, 5.97 (5.79) for NIV, and 1.46 (1.40) for MV. Thus, an unmeasured confounder would need to be associated with both OSA and death by a risk ratio of about 2 to fully explain the mortality association, whereas relatively weak confounding (risk ratio about 1.3) could explain the sepsis and AKI associations.

## Discussion

This nationally representative retrospective cohort study, among the largest analyses of OSA–pneumonia outcomes, identifies a bidirectional pattern of associations across nearly five million inpatient encounters: OSA was associated with greater acute respiratory failure and ventilatory support use, yet with lower in-hospital mortality and sepsis. Several mechanisms have been proposed for these findings, which we discuss below.

### Respiratory failure and noninvasive ventilation

The higher rate of acute respiratory failure in OSA patients (48.1% vs. 35.8%; aOR 1.56) may reflect the compounding of two forms of pulmonary dysfunction. OSA patients may operate at reduced ventilatory reserve: recurrent obstructive events may blunt hypercapnic and hypoxic ventilatory drive and produce baseline alveolar hypoventilation [8]. When pneumonia superimposes consolidation and ventilation-perfusion mismatch, these patients may cross the threshold into overt ventilatory failure at lower illness severity than their non-OSA counterparts. Microaspiration from upper airway collapsibility and coexisting gastroesophageal reflux may deliver a continuous inoculum of oropharyngeal pathogens to the distal airways [6], while OSA-associated disruption of the upper airway microbiome may heighten the inflammatory response to pneumonia [7]. The higher NIV use (aOR 3.26), the largest effect size in this study, may reflect this heightened failure burden. It may also be because the clinician threshold for initiating NIV is lower in patients with a known OSA diagnosis, and because patients with OSA may be more familiar with positive airway pressure interfaces.

### Interpreting the association with in-hospital mortality

The lower in-hospital mortality in OSA patients (aOR 0.74) despite greater comorbidity burden and higher respiratory failure rates is a notable finding of this study. Several non-mutually exclusive explanations warrant consideration, and our data cannot distinguish among them. One biological hypothesis is intermittent hypoxia-induced ischemic preconditioning: nightly hypoxia-reoxygenation cycles activate HIF-1α, VEGF, and Nrf2-responsive antioxidant programs (superoxide dismutase, catalase, heme oxygenase-1), which in experimental settings confer cytoprotection analogous to intermittent hypoxic conditioning [14]. Data from OSA patients with acute myocardial infarction, in which a higher apnea-hypopnea index was associated with lower peak troponin-T levels, have been interpreted as consistent with this hypothesis [14]. A second hypothesis is that chronic OSA induces immune remodeling toward anti-inflammatory phenotypes — M2 macrophage polarization, expanded regulatory T cells, and upregulated IL-10 [9, 15] — that could dampen the hyperinflammatory cascade in severe pneumonia. It is important to understand that these mechanisms are speculative and are derived largely from experimental or non-pneumonia studies.

Non-biological explanations are at least as plausible. Patients with a diagnosed OSA are engaged in care and may present earlier and receive faster antibiotic initiation. A 2025 Lancet Respiratory Medicine meta-analysis (1,175,615 patients) reported that CPAP therapy was associated with lower all-cause mortality (HR 0.63; 95% CI 0.56–0.72) [5], and a “healthy user” effect of structured healthcare engagement may contribute to the association observed here.

Similar patterns have been described in related contexts. In OSA–COPD overlap syndrome, PAP-treated patients have been reported to have better survival than untreated patients, and CPAP adherence has been associated with lower mortality [16, 17]. Yeh et al. (2024) reported an analogous pattern in OSA–COPD overlap patients undergoing CABG — higher rates of respiratory failure and AKI, but lower in-hospital mortality (aOR 0.53; 95% CI 0.35–0.81) versus COPD alone [18]. This convergence across acute settings is compatible with our hypotheses, but it is subject to the same limitations of observational and administrative data and does not establish that OSA-related adaptations are protective.

### Sepsis and septic shock

The lower rates of sepsis (aOR 0.89) and septic shock (aOR 0.80) in OSA patients are unexpected for a more comorbid population. One hypothesis is innate immune preconditioning: chronic OSA has been associated with M2 macrophage polarization, toll-like receptor desensitization, and Treg expansion [9, 10], which might attenuate the dysregulated inflammatory response underlying sepsis. Earlier hospital presentation and faster antibiotic initiation in patients with diagnosed OSA might also limit bacterial dissemination, and higher NIV use might reduce ventilator-associated pneumonia as a secondary sepsis driver. Our findings contrast with Huang et al. (2014), who reported higher sepsis mortality in a Taiwanese OSA cohort [19]; however, that study was substantially smaller (*n* = 168), used a different database, and did not restrict to pneumonia as the sepsis source.

### Acute kidney injury

The higher AKI rate in OSA patients (aOR 1.13) is coherent with established OSA–renal pathophysiology: chronic IH activates the renin-angiotensin-aldosterone system, promotes oxidative stress-mediated tubular inflammation, and may drive progressive renal vasculopathy [20, 21]. When pneumonia superimposes hemodynamic instability, systemic inflammation, and potential nephrotoxic antibiotic exposure onto this potentially compromised renal baseline — in a population in which 29.8% already had CKD — the risk of AKI may be increased.

### Clinical implications

Our findings are observational and hypothesis-generating, and they should be interpreted accordingly. Descriptively, patients with OSA hospitalized with pneumonia had higher rates of acute respiratory failure and NIV use, so this group may warrant closer respiratory monitoring. More broadly, our results identify OSA as a factor worth recording and examining in pneumonia outcome research, and prospective studies should examine whether adherence to CPAP and related factors in patients with OSA can reduce pneumonia risk or severity.

### Strengths and limitations

This study has several limitations. First, ICD-10-CM coding is subject to misclassification error; given that approximately 80% of moderate-to-severe OSA cases remain undiagnosed [1], many patients coded as non-OSA likely harbor unrecognized OSA, which may bias estimates toward the null. Second, the NIS lacks pneumonia severity scores (PSI/CURB-65), microbiological data, laboratory values, OSA severity (AHI), and CPAP adherence data, precluding causal inference. Third, the NIS captures discharges rather than individual patients; repeated admissions may contribute multiple encounters. Fourth, residual and unmeasured confounding from variables such as frailty, functional status, smoking history, and OSA phenotype persists despite multivariable adjustment. Fifth, because of the very large sample size, nearly all comparisons reached *p* < 0.0001, and several absolute differences (e.g., AKI 24.1% vs. 22.1%; septic shock 2.2% vs. 2.7%) were small; statistical significance should not be equated with clinical significance. E-values indicated that the sepsis and AKI associations could be explained by relatively weak unmeasured confounding. Finally, outcomes were identified by administrative codes without timing information, so the sequence of diagnoses within an admission is unknown.

Notwithstanding these limitations, the study has notable strengths. The NIS provides near-national coverage with nearly five million hospitalizations, offering a large sample size that yields important signals and generalizability across geographic regions, institution types, and payer categories. We also performed E-value analyses to quantify how strong unmeasured confounding would need to be to explain the observed associations. Unadjusted and adjusted estimates were directionally consistent across outcomes, although this does not address unmeasured confounding. The multidimensional outcome assessment extends prior single-outcome analyses, and the three-year study window captures real-world practice patterns. Some findings, especially the lower mortality in patients with OSA, are consistent with prior research [16–18]. Overall, many of the findings from this study can serve as hypothesis-generating signals for future research.

## Conclusion

In this nationally representative analysis, comorbid OSA in adults hospitalized with pneumonia was associated with higher rates of acute respiratory failure, ventilatory support, and AKI, and with lower in-hospital mortality and sepsis. These associations may reflect biological factors, such as hypoxic preconditioning and immune remodeling, and/or healthcare-related and coding factors. This hypothesis-generating study raises important questions, and future prospective studies should incorporate polysomnographic phenotyping, CPAP adherence data, pneumonia severity scoring, and microbiological profiles to generate more robust evidence.

## Supplementary Information

Supplementary Material 1.

## Data Availability

The Nationwide Inpatient Sample is publicly available from HCUP at https://hcup-us.ahrq.gov/nisoverview.jsp.
