# Supplementary material for: Association of obstructive sleep apnea with outcomes in hospitalized patients with pneumonia: a retrospective analysis of the nationwide inpatient sample
Source: Sleep Breath. 2026 Nov 12;30(6):311. doi: 10.1007/s11325-026-03855-0 (PMC13759686; doi:10.1007/s11325-026-03855-0)
Supplement: Supplementary file 1 [file 11325_2026_3855_MOESM1_ESM.docx]

**Impact of Obstructive Sleep Apnea on Hospitalized Patients with Pneumonia: A Retrospective Analysis of the Nationwide Inpatient Sample**

*Alam ABN, Iheme BC, Fatunmbi O, Adeyemi B, Baig S, Shayuk M*

**SUPPLEMENTARY MATERIAL**

**Supplementary Table S1. ICD-10-CM Codes Used for Case Identification, Exposure Classification, and Outcome Ascertainment**

This supplementary table lists all International Classification of Diseases, Tenth Revision, Clinical Modification (ICD-10-CM) diagnosis codes used in this study. Codes were applied to the 2018–2020 Nationwide Inpatient Sample (NIS) to identify: (1) the primary diagnosis of pneumonia qualifying a hospitalization for inclusion; (2) the index exposure of obstructive sleep apnea (OSA); (3) the primary outcome of in-hospital mortality (captured directly by the NIS discharge disposition field); and (4) all secondary outcomes and comorbidities included in multivariable regression models as outcomes or covariates. All codes were queried as secondary (any-listed) diagnoses unless otherwise specified.

| **Category / Condition** | **ICD-10-CM Code(s)** | **Official Code Description** | **Role in Study** |
| --- | --- | --- | --- |
| **SECTION 1 — Primary Diagnosis: Pneumonia (Inclusion Criterion — Principal Diagnosis Field)** | | | |
| **Viral pneumonia** | J12 | Viral pneumonia, not elsewhere classified | Primary DX |
|  | J12.0 | Adenoviral pneumonia | Primary DX |
|  | J12.1 | Respiratory syncytial virus pneumonia | Primary DX |
|  | J12.2 | Parainfluenza virus pneumonia | Primary DX |
|  | J12.3 | Human metapneumovirus pneumonia | Primary DX |
|  | J12.81 | Pneumonia due to SARS-associated coronavirus | Primary DX |
|  | J12.82 | Pneumonia due to coronavirus disease 2019 | Primary DX |
|  | J12.89 | Other viral pneumonia | Primary DX |
|  | J12.9 | Viral pneumonia, unspecified | Primary DX |
| **Pneumococcal pneumonia** | J13 | Pneumonia due to Streptococcus pneumoniae | Primary DX |
| **Haemophilus pneumonia** | J14 | Pneumonia due to Haemophilus influenzae | Primary DX |
| **Gram-negative pneumonia** | J15 | Unspecified bacterial pneumonia (gram-negative) | Primary DX |
|  | J15.0 | Pneumonia due to Klebsiella pneumoniae | Primary DX |
|  | J15.1 | Pneumonia due to Pseudomonas | Primary DX |
|  | J15.2 | Pneumonia due to staphylococcus | Primary DX |
|  | J15.20 | Unspecified staphylococcal pneumonia | Primary DX |
|  | J15.21 | Pneumonia due to MSSA | Primary DX |
|  | J15.211 | Pneumonia due to Methicillin susceptible Staphylococcus aureus | Primary DX |
|  | J15.212 | Pneumonia due to Methicillin resistant Staphylococcus aureus | Primary DX |
|  | J15.3 | Pneumonia due to streptococcus, group B | Primary DX |
|  | J15.4 | Pneumonia due to other streptococci | Primary DX |
|  | J15.5 | Pneumonia due to Escherichia coli | Primary DX |
|  | J15.6 | Pneumonia due to other Gram-negative bacteria | Primary DX |
|  | J15.61 | Pneumonia due to Acinetobacter baumannii | Primary DX |
|  | J15.69 | Pneumonia due to other Gram-negative bacteria | Primary DX |
|  | J15.7 | Pneumonia due to Mycoplasma pneumoniae | Primary DX |
|  | J15.8 | Pneumonia due to other specified bacteria | Primary DX |
|  | J15.9 | Unspecified bacterial pneumonia | Primary DX |
| **Other organism pneumonia** | J16 | Pneumonia due to other infectious organisms | Primary DX |
|  | J16.0 | Chlamydial pneumonia | Primary DX |
|  | J16.8 | Pneumonia due to other specified infectious organisms | Primary DX |
| **Pneumonia in diseases classified elsewhere** | J17 | Pneumonia in diseases classified elsewhere | Primary DX |
| **Unspecified pneumonia** | J18 | Pneumonia, unspecified organism | Primary DX |
|  | J18.0 | Bronchopneumonia, unspecified organism | Primary DX |
|  | J18.1 | Lobar pneumonia, unspecified organism | Primary DX |
|  | J18.2 | Hypostatic pneumonia, unspecified organism | Primary DX |
|  | J18.8 | Other pneumonia, unspecified organism | Primary DX |
|  | J18.9 | Pneumonia, unspecified organism | Primary DX |
| **SECTION 2 — Index Exposure: Obstructive Sleep Apnea (Any-Listed Secondary Diagnosis)** | | | |
| Obstructive sleep apnea | G47.33 | Obstructive sleep apnea (adult) (pediatric) | **Exposure (OSA group)** |
|  | G47.30 | Sleep apnea, unspecified | **Exposure (OSA group)ᵃ** |
|  | G47.31 | Primary central sleep apnea | Not included (excluded) |
|  | G47.32 | High altitude periodic breathing | Not included (excluded) |
|  | G47.34 | Idiopathic sleep-related nonobstructive alveolar hypoventilation | Not included (excluded) |
|  | G47.35 | Congenital central alveolar hypoventilation syndrome | Not included (excluded) |
|  | G47.36 | Sleep related hypoventilation in conditions classified elsewhere | Not included (excluded) |
|  | G47.37 | Central sleep apnea in conditions classified elsewhere | Not included (excluded) |
|  | G47.39 | Other sleep apnea | Not included (excluded) |
| **SECTION 3 — Primary Outcome: In-Hospital Mortality** | | | |
| In-hospital mortality | *NIS discharge disposition field* | Discharge disposition coded as "died" in NIS DISPUNIFORM variable (value = 20) | **Primary outcome** |
| **SECTION 4 — Secondary Outcomes (Any-Listed Secondary Diagnosis or Procedure Codes)** | | | |
| **Acute kidney injury (AKI)** | N17 | Acute kidney failure and acute kidney injury | Secondary outcome |
|  | N17.0 | Acute kidney failure with tubular necrosis | Secondary outcome |
|  | N17.1 | Acute kidney failure with acute cortical necrosis | Secondary outcome |
|  | N17.2 | Acute kidney failure with medullary necrosis | Secondary outcome |
|  | N17.8 | Other acute kidney failure | Secondary outcome |
|  | N17.9 | Acute kidney failure, unspecified | Secondary outcome |
| **Sepsis** | A40 | Streptococcal sepsis | Secondary outcome |
|  | A40.0 | Sepsis due to streptococcus, group A | Secondary outcome |
|  | A40.1 | Sepsis due to streptococcus, group B | Secondary outcome |
|  | A40.3 | Sepsis due to Streptococcus pneumoniae | Secondary outcome |
|  | A40.8 | Other streptococcal sepsis | Secondary outcome |
|  | A40.9 | Streptococcal sepsis, unspecified | Secondary outcome |
|  | A41 | Other sepsis | Secondary outcome |
|  | A41.0 | Sepsis due to Staphylococcus aureus | Secondary outcome |
|  | A41.01 | Sepsis due to MSSA | Secondary outcome |
|  | A41.02 | Sepsis due to MRSA | Secondary outcome |
|  | A41.1 | Sepsis due to other specified staphylococcus | Secondary outcome |
|  | A41.2 | Sepsis due to unspecified staphylococcus | Secondary outcome |
|  | A41.3 | Sepsis due to Hemophilus influenzae | Secondary outcome |
|  | A41.4 | Sepsis due to anaerobes | Secondary outcome |
|  | A41.50 | Gram-negative sepsis, unspecified | Secondary outcome |
|  | A41.51 | Sepsis due to Escherichia coli | Secondary outcome |
|  | A41.52 | Sepsis due to Pseudomonas | Secondary outcome |
|  | A41.53 | Sepsis due to Serratia | Secondary outcome |
|  | A41.54 | Sepsis due to Acinetobacter baumannii | Secondary outcome |
|  | A41.59 | Other Gram-negative sepsis | Secondary outcome |
|  | A41.81 | Sepsis due to Enterococcus | Secondary outcome |
|  | A41.89 | Other specified sepsis | Secondary outcome |
|  | A41.9 | Sepsis, unspecified organism | Secondary outcome |
| **Septic shock** | R65.21 | Severe sepsis with septic shock | Secondary outcome |
| **Acute respiratory failure** | J96.0 | Acute respiratory failure | Secondary outcome |
|  | J96.00 | Acute respiratory failure, unspecified whether with hypoxia or hypercapnia | Secondary outcome |
|  | J96.01 | Acute respiratory failure with hypoxia | Secondary outcome |
|  | J96.02 | Acute respiratory failure with hypercapnia | Secondary outcome |
| **Noninvasive ventilation (NIV)** | 5A09357 | Assistance with respiratory ventilation, less than 24 consecutive hours, continuous positive airway pressure (ICD-10-PCS) | Secondary outcome |
|  | 5A09457 | Assistance with respiratory ventilation, 24-96 consecutive hours, CPAP (ICD-10-PCS) | Secondary outcome |
|  | 5A09557 | Assistance with respiratory ventilation, >96 consecutive hours, CPAP (ICD-10-PCS) | Secondary outcome |
|  | 5A0935Z | Assistance with respiratory ventilation, <24h, bilevel (BiPAP) (ICD-10-PCS) | Secondary outcome |
|  | 5A0945Z | Assistance with respiratory ventilation, 24–96h, bilevel (BiPAP) (ICD-10-PCS) | Secondary outcome |
|  | 5A0955Z | Assistance with respiratory ventilation, >96h, bilevel (BiPAP) (ICD-10-PCS) | Secondary outcome |
| **Mechanical ventilation (MV)** | 5A1935Z | Performance of respiratory ventilation, <24h, continuous (ICD-10-PCS) | Secondary outcome |
|  | 5A1945Z | Performance of respiratory ventilation, 24–96h, continuous (ICD-10-PCS) | Secondary outcome |
|  | 5A1955Z | Performance of respiratory ventilation, >96h, continuous (ICD-10-PCS) | Secondary outcome |
| **SECTION 5 — Comorbidities Included as Covariates / Charlson Comorbidity Index Components (Any-Listed Secondary Diagnosis)** | | | |
| **COPD** | J44.0 | Chronic obstructive pulmonary disease with acute lower respiratory infection | Covariate |
|  | J44.1 | COPD with acute exacerbation | Covariate |
|  | J44.9 | Chronic obstructive pulmonary disease, unspecified | Covariate |
|  | J43 | Emphysema | Covariate |
|  | J41 | Simple and mucopurulent chronic bronchitis | Covariate |
|  | J42 | Unspecified chronic bronchitis | Covariate |
| **Type 2 diabetes mellitus** | E11 | Type 2 diabetes mellitus | Covariate |
|  | E11.0 | Type 2 DM with hyperosmolarity | Covariate |
|  | E11.1 | Type 2 DM with ketoacidosis | Covariate |
|  | E11.21 | Type 2 DM with diabetic nephropathy | Covariate |
|  | E11.36 | Type 2 DM with diabetic cataract | Covariate |
|  | E11.40 | Type 2 DM with diabetic neuropathy, unspecified | Covariate |
|  | E11.65 | Type 2 DM with hyperglycemia | Covariate |
|  | E11.9 | Type 2 DM without complications | Covariate |
| **Chronic kidney disease (CKD)** | N18 | Chronic kidney disease | Covariate |
|  | N18.1 | CKD, stage 1 | Covariate |
|  | N18.2 | CKD, stage 2 (mild) | Covariate |
|  | N18.3 | CKD, stage 3 (moderate) | Covariate |
|  | N18.30 | CKD, stage 3 unspecified | Covariate |
|  | N18.31 | CKD, stage 3a | Covariate |
|  | N18.32 | CKD, stage 3b | Covariate |
|  | N18.4 | CKD, stage 4 (severe) | Covariate |
|  | N18.5 | CKD, stage 5 | Covariate |
|  | N18.6 | End-stage renal disease | Covariate |
|  | N18.9 | CKD, unspecified | Covariate |
| **Chronic heart failure** | I50 | Heart failure | Covariate |
|  | I50.1 | Left ventricular failure, unspecified | Covariate |
|  | I50.20 | Unspecified systolic (congestive) heart failure | Covariate |
|  | I50.21 | Acute systolic (congestive) heart failure | Covariate |
|  | I50.22 | Chronic systolic (congestive) heart failure | Covariate |
|  | I50.23 | Acute on chronic systolic heart failure | Covariate |
|  | I50.30 | Unspecified diastolic (congestive) heart failure | Covariate |
|  | I50.31 | Acute diastolic heart failure | Covariate |
|  | I50.32 | Chronic diastolic heart failure | Covariate |
|  | I50.33 | Acute on chronic diastolic heart failure | Covariate |
|  | I50.40 | Unspecified combined systolic and diastolic heart failure | Covariate |
|  | I50.9 | Heart failure, unspecified | Covariate |
| **Obesity** | E66 | Overweight and obesity | Covariate |
|  | E66.0 | Obesity due to excess calories | Covariate |
|  | E66.01 | Morbid (severe) obesity due to excess calories | Covariate |
|  | E66.09 | Other obesity due to excess calories | Covariate |
|  | E66.1 | Drug-induced obesity | Covariate |
|  | E66.2 | Morbid (severe) obesity with alveolar hypoventilation | Covariate |
|  | E66.3 | Overweight | Covariate |
|  | E66.9 | Obesity, unspecified | Covariate |
| **Essential hypertension** | I10 | Essential (primary) hypertension | Covariate |
| **Atrial fibrillation** | I48.0 | Paroxysmal atrial fibrillation | Charlson CCI component |
|  | I48.11 | Longstanding persistent atrial fibrillation | Charlson CCI component |
|  | I48.19 | Other persistent atrial fibrillation | Charlson CCI component |
|  | I48.20 | Chronic atrial fibrillation, unspecified | Charlson CCI component |
|  | I48.91 | Unspecified atrial fibrillation | Charlson CCI component |
| **Cerebrovascular disease** | I63 | Cerebral infarction | Charlson CCI component |
|  | I69 | Sequelae of cerebrovascular disease | Charlson CCI component |
| **Peripheral vascular disease** | I70 | Atherosclerosis | Charlson CCI component |
|  | I73.9 | Peripheral vascular disease, unspecified | Charlson CCI component |
| **Chronic liver disease** | K70 | Alcoholic liver disease | Charlson CCI component |
|  | K74 | Fibrosis and cirrhosis of liver | Charlson CCI component |
|  | K76.0 | Fatty (change of) liver, not elsewhere classified | Charlson CCI component |
| **Malignancy** | C00–C26 | Malignant neoplasms, lip, oral cavity, pharynx, digestive organs | Charlson CCI component |
|  | C30–C39 | Malignant neoplasms, respiratory and thoracic organs | Charlson CCI component |
|  | C40–C41 | Malignant neoplasms of bone and articular cartilage | Charlson CCI component |
|  | C43–C96 | Melanoma and other malignant neoplasms | Charlson CCI component |
| **Dementia** | F01 | Vascular dementia | Charlson CCI component |
|  | F02 | Dementia in other diseases classified elsewhere | Charlson CCI component |
|  | F03 | Unspecified dementia | Charlson CCI component |
|  | G30 | Alzheimer disease | Charlson CCI component |
| **Connective tissue disease** | M05 | Seropositive rheumatoid arthritis | Charlson CCI component |
|  | M32 | Systemic lupus erythematosus | Charlson CCI component |
|  | M34 | Systemic sclerosis (scleroderma) | Charlson CCI component |
| **Peptic ulcer disease** | K25 | Gastric ulcer | Charlson CCI component |
|  | K26 | Duodenal ulcer | Charlson CCI component |
|  | K27 | Peptic ulcer, site unspecified | Charlson CCI component |
| **HIV/AIDS** | B20 | Human immunodeficiency virus (HIV) disease | Charlson CCI component |
|  | Z21 | Asymptomatic HIV infection status | Charlson CCI component |
| **SECTION 6 — Exclusion Criteria: Palliative and Hospice Care** | | | |
| Palliative care | Z51.5 | Encounter for palliative care | *Exclusion criterion* |
| Hospice care | Z51.5 | Palliative care encounter (NIS discharge status: hospice) | *Exclusion criterion* |
|  | NIS DISPUNIFORM = 50, 51 | Discharge to hospice (home or medical facility) — NIS discharge disposition field | *Exclusion criterion* |

*DX = diagnosis; ICD-10-CM = International Classification of Diseases, Tenth Revision, Clinical Modification; ICD-10-PCS = International Classification of Diseases, Tenth Revision, Procedure Coding System; NIS = Nationwide Inpatient Sample; OSA = obstructive sleep apnea; CKD = chronic kidney disease; COPD = chronic obstructive pulmonary disease; DM = diabetes mellitus; CHF = chronic heart failure; CCI = Charlson Comorbidity Index; MSSA = methicillin-susceptible Staphylococcus aureus; MRSA = methicillin-resistant Staphylococcus aureus; NIV = noninvasive ventilation; CPAP = continuous positive airway pressure; BiPAP = bilevel positive airway pressure; MV = mechanical ventilation.*

*ᵃ G47.30 (sleep apnea, unspecified) was included in the OSA exposure group in sensitivity analyses. The primary analyses used G47.33 exclusively.*

*All ICD-10-CM codes are those valid for the fiscal years 2018, 2019, and 2020 as published by the Centers for Medicare and Medicaid Services (CMS). Procedure codes for ventilatory support follow ICD-10-PCS notation used in NIS. Code ranges (e.g., J12–J18) indicate that all codes within the range and at all levels of specificity were queried.*

*The Charlson Comorbidity Index was calculated using the Quan et al. (2005) ICD-10 adaptation of the original Charlson index. Refer to: Quan H et al. Updating and validating the Charlson comorbidity index and score for risk adjustment in hospital discharge abstracts using data from 6 countries. Am J Epidemiol. 2011;173(6):676–682.*
